# Knowledge, Attitude and Practices (KAP) of Ruminant Livestock Farmers Related to Zoonotic Diseases in Elassona Municipality, Greece

**DOI:** 10.3390/ejihpe12030019

**Published:** 2022-02-25

**Authors:** Athanasios Moutos, Chrysoula Doxani, Ioannis Stefanidis, Elias Zintzaras, Georgios Rachiotis

**Affiliations:** 1 Department of Biomathematics, School of Medicine, University of Thessaly, Papakyriazi 22, 41222 Larissa, Greece; doxani@med.uth.gr (C.D.); zintza@med.uth.gr (E.Z.); 2 Department of Nephrology, School of Medicine, University of Thessaly, 41110 Larissa, Greece; stefanid@med.uth.gr; 3 Center for Clinical Evidence Synthesis, Tufts University School of Medicine, Boston, MA 02111, USA; 4 The Institute for Clinical Research and Health Policy Studies, Tufts Medical Center, Boston, MA 02111, USA; 5 Department of Hygiene and Epidemiology, Medical Faculty, School of Health Science, University of Thessaly, 41110 Larissa, Greece; grach@med.uth.gr

**Keywords:** knowledge, attitude, practice, zoonoses, Greece, ruminant livestock farmers

## Abstract

Zoonotic diseases represent a significant health and economic burden in countries that rely on small ruminant milk production, such as Greece. Greece is endemic for many zoonotic diseases, some of which have occupational determinants. Our aim was to evaluate knowledge, attitude, and practices of livestock ruminant farmers concerning zoonoses. This study was performed as a cross-sectional study, using a questionnaire. We interviewed ruminant farmers (*n* = 204) from 33 settlements of an area with intense agrarian activity. Three index variables, namely knowledge score, attitude score, and practice score, were constructed. The relations between the explanatory variables and the three indexes were assessed based on linear regression analyses. Regarding practices, 23 (11.3%) consume unpasteurized milk or products from unpasteurized milk and no one takes precautionary measures when assisting animals in parturition or during handling birth material. Education level was positively associated with better knowledge and practices, while close veterinary supervision of the farm was associated with better practices regarding the zoonoses prevention. The results indicate the need for continuous awareness and education actions. Close contact with a veterinarian can be utilized as a key tool both with the current brucellosis vaccination program and in the design of awareness campaigns regarding zoonoses in collaboration with other public health personnel.

## 1. Introduction

Small ruminant milk production is of high significance for developed Mediterranean countries, being an important portion of the rural income and boosting national economy [1]. Greece is among the leading countries with 840.140 tons of milk per year [2]. Zoonotic diseases have a tremendous impact on livestock production, public health, and, consequently, to the economy [3].

Almost 60% of currently known infectious diseases and up to 75% of emerging infectious agents are of zoonotic origin [4,5]. Zoonotic infections account for devastating epidemics, with the COVID-19 pandemic being the most notable. However, endemic and neglected zoonoses represent a more insidious and chronic treat for global health and national economies [6].

Many zoonoses are thought to be occupational health hazards [7]. Livestock farmers are at risk since different type and intensity of contacts may lead to zoonotic infections [8]. In the US and Italy, zoonotic agents are considered as an occupational hazard and health risk for livestock workers [9,10]. In Greece, in spite of many unreported cases, brucellosis remains an occupational disease [11].

Elassona area is an agrarian municipality of Larissa division consisting of 102.5 km^2^ with a human population of 32.121, the majority of which resides in rural areas [12]. In Elassona, 302.917 sheep, 96.907 goat, and 26.876 cattle [13] are reared under various traditional farming systems. The area has the highest per capita milk availability, with Larissa being the first Greek division in terms of small ruminant milk production [2], and a very high density of animals. Additionally, Elassona is located in the southwest of Olympus Mountain, is the home of a rich diversity of fauna.

Greece is endemic for many zoonotic diseases including brucellosis, Q fever, echinococcosis/hydatidosis, leishmaniasis, food-borne zoonoses, and others which have tremendous economic and public health consequences [14,15,16]. Larissa has the highest prevalence value of human brucellosis from the regions under the ruminants’ vaccination program [11]. In addition, a rabies case in a dog was confirmed in Elassona in February 2014, during the 2012–2014 outbreaks [17]. The extent of the connection among humans, domestic animals, and wildlife in this area reveals a high potential for zoonoses transmission.

Knowledge, Attitudes, and Practices (KAP) surveys provide crucial information to explore risk factors and potential intervention strategies for disease management. Hence, farmers’ behavior is strongly affected by their knowledge and attitude [18]. Poor knowledge of a disease correlates with disease prevalence and can fire a vicious cycle between underdiagnosis/underreporting and awareness deficit [19,20]. Studies from Egypt [21], Turkey [22], India [23], and other countries have mentioned the necessity for zoonotic diseases education and KAP surveys asset to grasp country-specific circumstances.

The objectives of this study are to assess the knowledge about zoonoses with high-risk potential for ruminant livestock keepers and identify attitudes and practices trends that are related to zoonotic disease transmission in the Greek rural areas. The information gathered can aid in effective policy development and guide local awareness programs as well as educational actions in the concept of one health medicine for the control of zoonotic diseases.

## 2. Materials and Methods

The protocol of this study was approved by the Steering Committee of the Post graduate program MSc Research Methodology in Biomedicine, Biostatistics and Clinical Bioinformatics, code: 3/10-9-2020. Informed written consent was obtained from all subjects involved in the study. The study was conducted according to the guidelines of the Declaration of Helsinki.

This study was performed as a cross-sectional study between November 2020 and February 2021.

### 2.1. Study Area

Municipality of Elassona consists of 52 settlements and belongs to Larissa division in Thessaly region [12]. The area of Elassona is dominated by small ruminant dairy farming. In addition, dairy and beef cattle keepers as well as small-scale household breeders mark the area as agricultural.

The target population was the livestock farmers of the 52 settlements. A total of 33 out of the 52 villages were selected based on convenience, defined as the interviewer performing veterinary practice concurrently, and purpose, to cover the whole area geographically. The sample size of 204 livestock farmers was selected conveniently with some snowball sampling also. Farmers’ profile is depicted on Table 1.

This area was selected because of the high density of animals, the history of brucellosis’ high prevalence, and the presence of endemic zoonotic diseases with high transmission potential. Moreover, the large-scale livestock operations and by extension the many immigrants working positions in these farms is an important feature of the area.

### 2.2. Questionnaire Design

A questionnaire consisted of 6 sections and 43 questions with several sub-questions, was developed in the Greek language. The questionnaire contained binary, multiple-selection, open-ended, and Likert-scale questions. Several of these questions were utilized in the construction of three index variables, namely knowledge, attitude, and practice score.

Demographic and farm-associated information included age, gender, education, years of farming practice, ownership of other domestic animals, rodent quantity on farm, and veterinary supervision. Knowledge questions were based on the endemic zoonoses history, and transmission potential. The questions that were used in the building of knowledge scores concerned the zoonotic agents: brucella, its hosts, symptoms, and abortifacient potential, toxoplasma, its hosts, and its abortifacient potential, echinococcus and rabies virus zoonotic potential and hosts. In addition, the participants were asked if proper heat processing of animal products protects from zoonoses and whether some microorganisms responsible for farm animals’ diarrhea are zoonotic. The last question was about the relation of ventilation and cleanliness within the farm with zoonoses transmission. The detailed questions are depicted in Table 2.

Attitudes and practices formation considered the historical problem of brucellosis in this area and health guidelines for zoonotic disease prevention. The questions used in the construction of attitude score were if animals died from disease should be buried and lime or other disinfectant have to be applied and whether animals have to be dewormed. For the building of practice score, the participants were asked if they consume unpasteurized milk or milk products, if they wash their hands after animal handling, if they wear gloves on farm tasks and especially when excoriating, if they wear special clothing on the farm, and if they investigate through laboratory testing for the cause of abortions. They were also asked if they wear protective equipment such as gloves when assisting animals in parturition and handling birth material, if they buy animals only from farms with known health status, if they quarantine and test for zoonotic agents the animals they buy, and if children have contact with the farm animals. Additionally, they had to answer if they smoke and if they consume food or drinks during farm tasks, and whether they have been vaccinated for tetanus in the last decade. The questions are depicted in Table 3. Finally, a medical history section for zoonotic infection was included.

Before the onset of the interviews, the objectives of the study, willingness of results communication after the end of the study, and anonymity were explained to the livestock farmers and an informed consent document was filled.

### 2.3. Statistical Analysis

The questionnaires were checked for completeness before entering the data into IBM SPSS Statistics for Windows Version 25.0 statistical software (released 2017). There were scarce missing replies, because the interviews were performed face-to-face by a trained veterinarian, and these were identified as missing data in the analysis.

We used the previous mentioned demographic and farm characteristics as explanatory variables. A knowledge score (range 0–7) was prepared adding up farmer’s knowledge regarding specific questions and sub-questions. A score 1.0 was awarded if the participant could choose the correct answer and no score granted for an incorrect reply. For question 1 the score 1.0 was awarded if the farmer could identify Brucella as a zoonotic agent, at least all its ruminant hosts, three brucellosis symptoms, and its abortifacient potential for a pregnant woman, at the same time. In this rationale, a score of 1.0 was awarded if the participant could identify the zoonotic agent and at least one primary host for questions 2, 3, and 4. The attitude score (range 0–2) and the practice score (range 0–13) followed the same score system. The data were then entered into SPSS and descriptive analyses were carried out.

The unconditional association between each explanatory variable and knowledge score was searched through a series of univariable linear regression analyses. After the preliminary analysis with knowledge score as the outcome variable, the same series of univariable analyses was followed with attitude score as the outcome variable, including knowledge score with the previous independent variables this time. Lastly, the same univariable analyses with practice score as the outcome variable and knowledge and attitude scores as independent variables together with the previous explanatory variables were made. The explanatory variables with *p*-value < 0.25 were selected for inclusion in the multivariable model building.

A forward stepwise method of multivariable linear regression analysis was performed for each of the three scores, by adding foremost the variable that had the smallest *p*-value in the univariable analyses. Explanatory variables with *p*-value < 0.05 were retained in the final model. The adjusted R2 was used to assess how well the model accounts for the outcome of the data. The internal consistency of the questionnaire was assessed by calculating the alpha Cronbach.

## 3. Results

### 3.1. Sociodemographic and Farming Characteristics

A total of 204 ruminant farmers were interviewed during the study period, with a 93% response rate. The mean age of participants was 49.36 years (range 18–77 and standard deviation 13.57) and 83.3% were male. In terms of education, 30.9% reported some level of primary school attendance and 8.8% had completed university or college education. A total of 59.8% of participants were sheep farmers, while 10.8% breed mixed ruminant species, either sheep and goats or cattle and small ruminants. The veterinary supervision was further divided into two categories for the statistical analyses because only one farmer reported no veterinary attendance on farm and one indicated constant supervision. The detailed profile of the participants is presented in Table 1.

### 3.2. Knowledge on Zoonotic Diseases

Out of the 204 participants, 201 had heard the term ‘zoonoses’, but all of them could understand the meaning when explained. Besides, the detailed information used in the building of knowledge score that is mentioned in Table 2, the respondents were asked if they knew the zoonotic potential of some microbe agents. Of the farmers, 127 (62.3%) were aware of the zoonotic nature of mycobacterium tuberculosis, 161 (78.9%) about anthrax, 163 (79.9%) about salmonella, 112 (54.9%) about west Nile virus, 70 (34.3%) about tetanus, 79 (38%) about H1N1 virus, 30 (14.7%) about leptospira, 10 (4.9%) about E. coli strains, 3 (1.5%) about cryptosporidium, 1 (0.5%) about coxiella (Q fever), and campylobacter. Regarding dermatitis problems, 22.1% of the farmers could identify the zoonotic potential of some agents, mainly dermatofytes, and hardly anyone had heard of orf virus’ ability to colonize human skin.

### 3.3. Attitude and Practices on Zoonotic Diseases

Among the farmers, 181 (88.7%) believed that an animal that died because of a disease should be buried and disinfectants should be used, and 195 (95.6%) replied that animals must be dewormed for zoonotic disease prevention. Nevertheless, all of them admitted that in practice they usually do not bury dead animals with disinfectants, and they omit regular deworming of their domestic pet animals. Additionally, 85.3% considers from very possible to absolutely certain that a future infection from their animals will occur, 90.2% finds the implementation of the brucellosis vaccination program from very good to perfect, and 88.2% believes that it can lead to the extinction of the disease. Some objections concern the illegal animal trafficking, vaccine ineffectiveness, possible incomplete vaccination of some flocks, and inadequate veterinary service due to staff deficit.

Regarding practices, 23 (11.3%) consume raw milk or products from raw milk despite the brucellosis problem until today in the area, 152 (74.5%) smoke during farm work, 195 (95.6%) consume food or drinks, mainly coffee, while working on farm, only 7 (3.4%) quarantine and test the animals they buy for zoonotic diseases, and no one takes precautionary measures when assisting animals in parturition or in the handling of birth material. Moreover, 63 (30.9%) had been vaccinated for tetanus the last decade, but it is questionable whether some participants could differentiate between a tetanus serum infusion after an injury and a tetanus vaccine. The detailed information and questions involved in the construction of attitude and practice scores are depicted in Table 3. Cronbach’s alpha was calculated at 0.79 for the knowledge section of the questionnaire, and the overall Cronbach (knowledge, attitude, practice sections) was estimated at 0.73.

### 3.4. Medical History on Zoonotic Diseases

This sector’s findings confirm the area’s high zoonotic dynamics, with 29.4% of farmers having been infected from at least one zoonotic disease. Thoroughly, out of the 204 farmers, 54 (24.5%) have been infected from brucella, 2 (1%) from anthrax, 2 (1%) from both brucella and anthrax, 1 (0.5%) from E. coli strains, and 1 (0.5%) have suffered dermatitis, most possibly due to dermatophytes. Farmers having suffered a zoonotic disease reported 112.7 mean days for full recovery (range 6–700) and 18.9 mean days out of labor (range 0–180), while 6 (2.9%) reported having acquired permanent complications from the disease.

### 3.5. Univariable Analyses

Increase in farmer’s age (*p* < 0.001) and years of livestock farming (*p* < 0.001), having a significant correlation (Pearson coefficient = 0.835), were negatively associated with the zoonotic knowledge score. On the contrary, a higher education level (*p* < 0.001) and a more consistent veterinary supervision (*p* = 0.005), were found to be positively associated with the zoonoses knowledge. Concerning attitude score, age (*p* = 0.018) was negatively associated, and knowledge score (*p* < 0.001) was positively associated. In addition, age (*p* < 0.001) and years of livestock farming (*p* = 0.001) were also negatively associated with practice score. Moreover, higher education level (*p* < 0.001), more frequent veterinary supervision (*p* = 0.003), and a better knowledge score (*p* = 0.005) were positively associated with the practice score. The detailed results of univariable analyses are displayed in Table 4.

### 3.6. Multivariable Analyses

The education level (*p* = 0.001), which was positively associated with the zoonotic disease knowledge score, and the years of livestock farming (*p* = 0.038), which was negatively associated, were the significant parameters. Regarding attitude score, knowledge score (*p* < 0.001) was the only important parameter, positively correlated. Lastly, the education level (*p* < 0.001) and the degree of veterinary supervision (*p* = 0.032), both positively associated, were retained in the final model for practice score. The detailed results of multivariable analysis are presented in Table 5. We can conclude that education level is a significant factor and higher education is correlated with better knowledge and practices. On the other hand, the increase in farmers’ years of farming correlates with poor knowledge of zoonotic diseases. Another crucial factor is the close veterinary supervision of the farm, which results in adoption of better practices regarding zoonosis prevention.

## 4. Discussion

In this study, we made the first attempt in Greece to assess the knowledge, attitude, and practices of ruminant farmers regarding zoonotic diseases in an area of high agricultural interest. The results indicate that, despite of an overall good knowledge about specific zoonotic diseases used in the construction of knowledge score, there are some serious knowledge and awareness shortcomings.

One of the most important findings is the identification of several high-risk practices, with negligence of protective equipment while assisting an animal’s parturition and handling birth material, being universal among the participants. Globally, brucellosis KAP studies have revealed high-risk activities such as the handling of birth material without protective equipment and consumption of cheese from unpasteurized milk in Egypt [21] and in Jordan [24]. In our study, the consumption of unpasteurized milk or products from unpasteurized milk is not factoid given the brucellosis situation in Elassona area [11].

Furthermore, common practices such as the introduction of animals to farms from flocks of unknown health status without testing and quarantine can possibly explain the existence of brucellosis problem until today. The high-risk self-reported practices imply a knowledge gap towards zoonotic disease transmission or/and disease prevalence. A similar pattern of increased awareness due to high endemicity and high-risk practices due to knowledge lack of the transmission modes was found in Egypt [25].

Brook and McLachlan [26] indicated that the level of disease awareness among farmers in North America is associated to prevalence of the disease. In our study, the zoonotic nature of some agents as coxiella, cryptosporidium, and E. Coli remains unknown for the majority of the participants, despite of an important documented prevalence in ruminant farms [27,28,29]. This can possibly be explained because of the low number of human infections until today or many unreported cases due to low disease severity.

The education of farmers has been associated with better zoonotic disease knowledge and practices. In addition to this, the increase in years of farming correlates with poor knowledge about zoonosis. These findings can be attributed to the improvement of education system across the years, the acquaintance of the new generation with technological developments and the introduction of training courses for the education of new farmers in this field. Such training courses can play a more active role in zoonotic disease awareness, particularly for the older farmers.

Moreover, the current brucellosis vaccination and testing program in the Elassona area represents a good opportunity to promote zoonotic diseases awareness and to communicate helpful information, especially in the crucial part of zoonotic disease prevention. Studies from South Africa [30] and India [31] have shown that veterinary consultation plays a crucial role in farmer’s knowledge about brucellosis. Additionally, the absence of collaboration with a veterinarian has also been linked with knowledge gaps and risky practices about bovine brucellosis in Portugal [32]. Our findings indicate that veterinary supervision is associated with better practices for prevention of zoonotic diseases. The role of the veterinarian in the communication of knowledge and training of farmers seems to be very crucial for the public health.

Our study has several limitations, as an observational and cross-sectional study. Given the cross-sectional nature of the current survey it is impossible for us to document causal relationships. Our study was questionnaire-based and there is a potential for recall bias to occur. The response bias is inevitable due to the utilization of multiple-choice answers, although it might have been mitigated in the construction of knowledge score because of some strict requirements. Attitude replies clearly do not correspond to practices, because many farmers admitted that despite of knowing the proper action, they do not implement it either due to cost or to handwork required. This is further comprehensible considering the high risky behaviors uncovered through the self-reported practices. Furthermore, the findings have weak external validity (generalizability) because of convenience and local sampling. Nevertheless, the Cronbach’s alpha results, suggest that our questionnaire demonstrated a good internal consistency.

## 5. Conclusions

Conclusively, our study highlighted the specific areas in Greek ruminant farmers KAPs that must be utilized for design of zoonotic disease awareness and prevention programs. Education and veterinary supervision of the farm are two significant factors that must be in the core of any program oriented towards the improvement of the zoonotic disease KAPs of the farmers.

Similar KAP studies could be used for the rest of the country, with priority to regions of similarly intense agrarian activity, to establish baseline KAP for different agricultural communities. Afterwards, the gathered information could be utilized to design purposeful awareness and education campaigns. Specific educational activities should be launched with focus on older farmers. The cooperation between veterinarians and public health officers is essential to design and implement an education program for farmers under the prism of one health approach.

## Figures and Tables

**Table 1:** Frequency table for demographic and farm variables, in a KAP study relating to zoonotic diseases, among livestock owners/farmers in Greece.

Variable	Category	Number (%)
Age	Mean	49.36
	Minimum	18
	Maximum	77
	St. deviation	13.57
Gender	Male	170 (83.3)
	Female	34 (16.7)
Education level	Primary school	63 (30.9)
	High school	43 (21.1)
	Senior high school	80 (39.2)
	University/College	18 (8.8)
Years of livestock farming	Mean	29.49
	Min	3
	Max	58
	St. deviation	14.83
Main ruminant species farming	Goats	22 (10.8)
	Sheep	122 (59.8)
	Cattle	38 (18.6)
	Mixed species farming	22 (10.8)
Owns other productive animals	Poultry	130 (63.7)
	Swine	26 (12.7)
	Other	80 (39.2)
Owns other domestic animals/pets	Dogs	202 (99)
	Cats	185 (90.7)
	Horses	45 (22.1)
	Other	5 (2.5)
Veterinary supervision	None	1 (0.5)
	Small	129 (63.2)
	Often	73 (35.8)
	Constant	1 (0.5)

**Table 2:** Frequency table for knowledge score answers of livestock farmers relating to zoonotic diseases in Elassona area, Greece.

Variable	Response	Number (%)
Knowledge		
1.a. Do you know the zoonotic agent brucella?	Yes	204 (100)
	No	0 (0)
1.b. Which of the following species are brucella hosts?	Sheep	204 (100)
	Goats	203 (99.5)
	Cattle	187 (91.7)
	Swine	9 (4.4)
	Dog	5 (2.5)
1.c. Which of the following are human brucellosis symptoms?	Fever	202 (99)
	Perspiration	204 (100)
	Fatigue/Weakness	204 (100)
	Myalgia/Muscle pain	197 (96.6)
	Backpain	186 (91.2)
	Orchitis	159 (77.9)
	Pneumonia	54 (26.5)
	Hepato/Splenomegaly	1 (0.5)
	Other	0 (0)
1.d. Can brucellosis become a problem to a woman’s gestation?	Yes	197 (96.6)
	No	7 (3.4)
2.a. Do you know the zoonotic agent toxoplasma and its hosts?	Yes	87 (42.6)
	No	117 (57.4)
2.b. Can toxoplasma become a problem to a woman’s gestation?	Yes	102 (50)
	No	102 (50)
3.a. Do you know the zoonotic agent echinococcus?	Yes	186 (91.2)
	No	18 (8.8)
3.b. Can echinococcus be transmitted from dogs to humans?	Yes	183 (89.7)
	No	21 (10.3)
3.c. Can echinoccocus be transmitted to humans from other animals?	Yes	124 (60.8)
	No	80 (39.2)
4.a. Do you know the zoonotic agent rabies virus?	Yes	204 (100)
	No	0 (0)
4.b. Can rabies virus be transmitted from dogs to humans?	Yes	204 (100)
	No	0 (0)
4.c. Can rabies virus be transmitted to humans from other animals?	Yes	200 (98)
	No	4 (2)
5. Can proper heat processing of animal products protect you from zoonotic diseases?	Yes	201 (98.5)
	No	3 (1.5)
6. Can some of the microorganisms responsible for farm animals’ diarrhea be transmitted to humans?	Yes	26 (12.7)
	No	178 (87.3)
7. Do you believe ventilation and cleanliness within a farm is related with zoonotic diseases transmission?	Yes	196 (96.1)
	No	8 (3.9)

**Table 3:** Frequency table for attitude and practices of livestock farmers relating to zoonotic diseases in Elassona area, Greece.

Variable	Response	Number (%)
Attitude		
1. Do you believe that an animal that died with disease should be buried and covered with lime or other disinfectant?	Yes	181 (88.7)
	No	23 (11.3)
2. Do you believe animals should have to be dewormed?	Yes	195 (95.6)
	No	9 (4.4)
Practices		
1. Do you consume unpasteurized milk or products from unpasteurized milk?	Yes	23 (11.3)
	No	181 (88.7)
2. Do you wash your hands after having contact with animals?	Yes	196 (96.1)
	No	7 (3.4)
3. Do you wear gloves during farm tasks?	Yes	66 (32.4)
	No	138 (67.6)
4. Do you wear special clothing on the farm?	Yes	192 (94.1)
	No	12 (5.9)
5. Do you wear gloves while excoriating?	Yes	133 (65.2)
	No	71 (34.8)
6. Do you investigate through laboratory testing for the causative agent of abortions?	Yes	4 (2)
	No	200 (98)
7. Do you wear protective equipment such as gloves when assisting animals in parturition and handling birth material?	Yes	0 (0)
	No	204 (100)
8. Do you buy animals only from farms with known health status?	Yes	17 (8.3)
	No	187 (91.7)
9. Do you quarantine and test the animals you buy for zoonotic agents?	Yes	7 (3.4)
	No	197 (96.6)
10. Do children have contact with your farm animals?	Yes	161 (78.9)
	No	43 (21.1)
11. Do you smoke while working on the farm?	Yes	152 (74.5)
	No	52 (25.5)
12. Do you consume food or drinks during farm tasks?	Yes	195 (95.6)
	No	9 (4.4)
13. Have you been vaccinated for tetanus in the last decade?	Yes	63 (30.9)
	No	141 (69.1)

**Table 4:** Univariable linear regression analysis, demonstrating the influence of explanatory variables on the outcome variables.

Variable	b	Adjusted R^2^	*p*-Value
Dependent variable: Knowledge score			
Age	−0.022	0.113	<0.001
Gender (male versus female)	0.253	0.006	0.13
Education level	0.343	0.144	<0.001
Years of livestock farming	−0.021	0.116	<0.001
Main ruminant species farming	0.125	0.008	0.107
Swine farming	0.361	0.014	0.053
Birds farming	−0.001	−0.005	0.991
Owns dog(s)	−0.124	−0.005	0.845
Owns cat(s)	0.416	0.014	0.051
Owns horse(s)	0.029	0.000	0.847
Veterinary supervision	0.363	0.034	0.005
Dependent variable: Attitude score			
Age	−0.005	0.022	0.018
Gender (male versus female)	0.082	0.001	0.292
Education level	0.061	0.017	0.036
Years of livestock farming	−0.003	0.011	0.077
Main ruminant species farming	0.034	−0.001	0.353
Swine farming	0.092	0.001	0.294
Birds farming	0.051	−0.001	0.403
Owns dog(s)	−0.158	−0.004	0.592
Owns cat(s)	0.117	0.002	0.242
Owns horse(s)	0.087	0.003	0.214
Veterinary supervision	0.055	0.004	0.362
Knowledge score	0.163	0.117	<0.001
Dependent variable: Practice score			
Age	−0.022	0.057	<0.001
Gender (male versus female)	−0.053	−0.005	0.818
Education level	0.352	0.078	<0.001
Years of livestock farming	−0.018	0.045	0.001
Main ruminant species farming	−0.079	−0.002	0.463
Swine farming	0.364	0.005	0.155
Birds farming	0.198	0.001	0.266
Owns dog(s)	0.223	−0.005	0.798
Owns cat(s)	0.214	−0.002	0.467
Owns horse(s)	0.301	0.006	0.143
Vet supervision	0.523	0.038	0.003
Knowledge score	0.266	0.033	0.005
Attitude score	0.030	0.000	0.884

**Table 5:** Multivariable linear regression analysis, demonstrating the influence of explanatory variables on the outcome variables.

	St. b	Adjusted R^2^	*p*-Value
Dependent variable: Knowledge score		0.158	
Education level	0.245		0.001
Years of livestock farming	−0.010		0.038
Dependent variable: Attitude score		0.117	
Knowledge score	0.163		<0.001
Dependent variable: Practice score		0.095	
Education level	0.310		<0.001
Veterinary supervision	0.374		0.032

## Data Availability

The dataset generated and analyzed in the current study is available from the corresponding author upon reasonable request.
